# Application of the health action process approach model for reducing excessive internet use behaviors among rural adolescents in China: a school-based intervention pilot study

**DOI:** 10.1186/s12889-021-10999-z

**Published:** 2021-05-26

**Authors:** Chengmeng Tang, Hein Raat, Mingxia Yan, Qiang Zhang, Kehan Li, Min Jiang, Wanjie Tang, Jiayi Chen, Ying Zhao, Qiaolan Liu

**Affiliations:** 1 Department of Health Behavior and Social Medicine, West China School of Public Health and West China Fourth Hospital, Sichuan University, Chengdu, China; 2 Department of Public Health, Erasmus MC - University Medical Center Rotterdam, Rotterdam, The Netherlands; 3 Department of Epidemiology and Health Statistics, West China School of Public Health and West China Fourth Hospital, Sichuan University, Chengdu, China; 4 Centre for Educational and Health Psychology, Sichuan University, Chengdu, China

**Keywords:** Health action process approach model, Internet use behavior, Rural adolescent, China

## Abstract

**Objective:**

There are few studies regarding Internet use behaviors of Chinese rural adolescents based on behavioral theory. The aim of this study is to examine the applicability and effectiveness of the health action process approach model (HAPA) in the intervention of excessive Internet use behaviors among rural adolescents in China.

**Methods:**

Three hundred twenty-seven participants who met the excessive Internet use criteria were involved in this study. Four interventions based on the HAPA model were conducted during 2015–2017. The structural equation model (SEM) was applied to fit the HAPA model.

**Results:**

The rate of average daily time spent online on weekends more than 4 h dropped from 57.2 to 39.1% (*P* < 0.001). The rate of daily game time more than 2 h decreased from 51.1 to 35.2% (*P* < 0.001). The result of SEM showed that both the applicability and effectiveness of the HAPA model were well in the intervention of excessive Internet use behaviors with good fitted indicators (χ^2^/df = 2.066, GFI = 0.889, CFI = 0.938, TLI = 0.928, IFI = 0.938, RMSEA = 0.057). The direct and indirect effects of the main pathways in the HAPA model were statistically significant (*P* < 0.05). The comparison analysis of HAPA model variables identified that outcome expectancy, intention, maintenance self-efficacy had been improved significantly after interventions.

**Conclusion:**

The intervention measures based on the HAPA model can effectively reduce excessive Internet use behaviors of Chinese rural adolescents, mainly through strengthen outcome expectancy, intention, and maintenance self-efficacy.

**Supplementary Information:**

The online version contains supplementary material available at 10.1186/s12889-021-10999-z.

## Introduction

The Internet increasingly has become one of the ways for adolescents to learn, as well as to entertain [1]. According to data released by the China Internet Network Information Center (CNNIC) in 2020, the number of users below the age of 18 years has been growing steadily; the Internet coverage rate among children and adolescents in rural areas in 2019 was 90.3%, which is almost the same rate as in urban areas (93.9%) [2]. The Internet may provide adolescents with more chances to acquire knowledge, and to communicate with others [1, 3], and even become an important medium for maintaining and consolidating friendship [4]. However, excessive Internet use may have a negative impact on the physical and mental health of adolescents [5–7]. Excessive Internet use is a behavior that is more prone to addiction, mainly manifested as a loss of control over Internet use [8]. Excessive use of Internet may be associated with visual fatigue and with the risk to develop myopia; it is also associated with sedentary behavior, which is a risk factor of obesity and cardiovascular diseases [7, 9]. Previous studies have shown that adolescents who spend relatively more time online tend to acquire more information about health risk behaviors such as smoking and drinking alcohol, but on the other hand tend to adopt such health risk behaviors through the Internet [4]. Moreover, adolescents who use the Internet relatively more often and longer, were shown to have more often problems with sleep disorders, depression, anxiety, and suicide [7, 10].

Exploring the influencing factors of adolescents’ Internet use behaviors is crucial for the development of targeted interventions. Previous studies have found that age [4, 11, 12], gender [4, 11, 13], academic pressure [4, 14, 15], and parental restrictions [6, 16] are related to adolescents’ Internet use behaviors. However, social-cognitive factors of adolescents were not studied well, such as self-efficacy, outcome expectancy, and intention, et al.; so far, most studies paid more attention to sociodemographic characteristics and social factors about family and peers [17]. As a consequence, few interventions to reduce excessive Internet use behaviors of adolescents are based on social-cognitive theories. A meta-analysis identified that interventions based on such theories, like cognitive-behavioral therapy (CBT) and peer support services, have positive effects when applied to Internet-addicted adolescents [18]. This suggests that to prevent addictive Internet use, it is also relevant to explore the social-cognitive factors influencing Internet use behaviors of normal adolescents.

Some evidence suggests that behavior interventions based on theoretical frameworks are more effective than interventions that lack a theoretical basis [19]. Social-cognitive factors were included in several behavioral change theories, one of which is the health action processes approach model (HAPA). The HAPA model combines the stages of change model with the continuous change model to explain the emergence and maintenance of behaviors, as well as related processes from the perspective of motivation and volition [20, 21]. According to the HAPA model, there are two phases before a behavior occurs: the motivational phase and the volitional phase. In the motivational phase, the behavior intention is determined by risk perception, outcome expectancy, and action self-efficacy. The volitional phase mainly includes planning and maintenance self-efficacy [20, 21].

The HAPA model has been used to study the determinants of behavior, as well as for the development of behavior change interventions in many studies; examples are studies with regard to physical activities and eating behaviors [20]. Studies show that the HAPA model can explain food safety behaviors very well [22]. And, for example, a study employing the HAPA model as a theoretical framework for an intervention to target sedentary behavior of college students was successful to reduce the frequency of sedentary behaviors [23]. Pinidiyapathirage et al. illustrated that the HAPA model is useful to develop effective interventions to promote physical exercise of women with a history of gestational diabetes [24].

To date, only very few studies addressed the determinants of excessive Internet use by adolescents in rural areas [13, 25, 26]; most studies focus on populations in urban areas [5, 27–31] . However, since the Internet coverage in rural areas has reached similar levels as in urban areas there is an urgent need to study the determinants of excessive Internet use of adolescents in rural areas to inform the development of effective interventions to promote ‘healthy’ Internet use by adolescents in rural areas. The HAPA model is expected to be very relevant to study this topic. Therefore, the objectives of this study include (1) examining the applicability of the HAPA model in Internet use behaviors; (2) examining the effectiveness of interventions based on the HAPA model in reducing excessive Internet use among Chinese rural adolescents.

## Materials and methods

### Participants

The participants were derived from the first, third, and fifth survey data of a 2-year longitudinal study for rural adolescents in Sichuan Province (2015–2017). Typical sampling and cluster sampling were used to select the study areas and participants in November 2015. In the first stage, a typical sampling method was used to select Zizhong County as the study area, representing rural areas in Sichuan. In the second stage, cluster sampling was used to randomly select two middle schools in the study area, one as the control school and the other as the experimental school. According to the characteristics of the Chinese middle school system and the 2-year longitudinal intervention design, only seventh grade and tenth grade students could meet the requirements. Therefore, all students in seventh grade and tenth grade in both schools were included in this study. After the first baseline survey, a follow-up survey was conducted every 6 months with a total of five surveys. In the fifth survey, the samples were in the nine-grade and twelve-grade. 1044, 973, and 874 samples were obtained in the experimental school in the first, third, and fifth survey, respectively, and 1399, 1777, 1583 samples respectively in the control school.

According to the baseline survey, samples who met one of the inclusion criteria were considered to have excessive use of the Internet and were involved in this study. The inclusion criteria were (1) average daily online time from Monday to Friday≥2 h (hour); (2) average daily online time on weekends≥4 h; (3) usually daily game time ≥ 2 h; (4) being online overnight at least once in the past 30 days. There were 327 eligible participants from the experimental school and 448 from the control school, divided into the experimental group and the control group respectively. The experimental and the control group were homogenous in sociodemographic characteristics (Appendix Table 1). The participants in the experimental group were enrolled in the current analysis.

### Intervention measures

After the baseline, four waves of intervention were conducted on the participants in the experimental school and each wave intervention was conducted before each follow-up survey (Table 1). The purpose of the first intervention was to cultivate the awareness and willingness of participants about Internet use behaviors. The main contents embraced publicizing the harms caused by excessive Internet use and the benefits of reducing Internet use time, with the objective of promoting the action self-efficacy about Internet use behaviors. Health education courses and intervention manuals were the primary methods. The second intervention aimed to consolidate the effect of the first intervention and to promote the formation of the intention of Internet use behavior. The professional guidance on plan formulation was added to the third intervention while strengthening the previous intervention contents. The main purpose of the fourth intervention was to strengthen the maintenance self-efficacy to improve Internet use behaviors. The principal method, health education courses with specific cases, aimed to build up confidence via ameliorating Internet use behaviors. Moreover, freely available table tennis rackets and other sports equipment were provided to encourage participants to take part in outdoor activities, thereby prompting them to cut down the online time.

**Table 1:** The contents of four interventions

Time	Aims	Methods	Implement and contents	HAPA variables basics
The first intervention	To cultivate the awareness and willingness of participants about Internet use behaviors	(1) Health education courses	**Implement:** Health education courses were conducted in class units and were taught by trained teachers who majored in health education. Each course lasted for 45 min for every class in every intervention. It was carried out by a combination of teacher teaching, videoes and scenario simulation. The courseware used in this intervention was copied to the school teachers, and they repeated the intervention contents to the participants in the daily class meetings. **Contents:** The main contents embraced publicizing the harms caused by excessive Internet use and the benefits of reducing Internet use time, with the objective of promoting the action self-efficacy about Internet use behaviors.	Risk perception Outcome expectancy Action self-efficacy
		(2) Customized manuals	**Implement:** The manuals were uniformly produced by the research team and distributed to all participants after the health education courses. **Contents:** The main contents were similar to the content of the health education courses, including the harms caused by excessive Internet use, the benefits of reducing Internet use time, and the correct approaches to surf online.	
The second intervention	To consolidate the effect of the first intervention and to promote the formation of the intention.	Health education courses	**Implement:** The implementation method was the same as the first intervention. **Contents:** The content emphasized and deepened the content of the first time.	Risk perception Outcome expectancy Action self-efficacy Intention
The third intervention	To help participants formulating plans.	(1) Health education courses	**Implement:** The implementation method was the same as the first intervention. **Contents:** The main contents were how to make plans to help the participants to turn their intention into actual behaviors, including the duration, the implementation of the plan, etc.	Planning
		(2) Guidance on plan formulation	**Implement:** After the health education courses, one-to-one guidance was provided for the participants by the trained teachers. **Contents:** The guidance content was about clarifying the plan’s objectives, implementation methods, duration, possible obstacles, obstacle solutions, etc.	
The fourth intervention	To strengthen the maintenance self-efficacy and reduce the online time.	(1) Health education courses	**Implement:** The implementation method was the same as the first intervention. **Contents:** The main contents were the introduction of specific cases (including successful cases and failure cases of reducing online time) and analysis of the reasons for success or failure.	Maintenance self-efficacy
		(2) Provide physical exercise equipment	Provided freely table tennis rackets, skipping ropes, and other sports equipment to every class.	Internet use behaviors

### Measures

#### Sociodemographic characteristics

Sociodemographic characteristics included age, gender, grade, and left-behind status. The left-behind status was referred to adolescents aged 17 years or younger who have been left in their rural communities by one or both parents migrating in search of work in cities.

#### Internet use behaviors

Internet use behaviors included four items: (1) “Your average daily Internet time from Monday to Friday (included smartphone and computer)”, the answers were scored 1–5, representing “≥4 h”, “3 h”, “2 h”, “1 h”, and “almost not”, respectively; (2) “Your average daily Internet time on weekends (included smartphone and computer)”, the answers were scored 1–5, representing “≥9 h”, “7–8 h”, “4–6 h”, “2–3 h”, and “≤1 h”, respectively; (3) “Your usually daily game time (included game devices, hand-held game devices, smartphone and computer)”, the answers were ranked 1–6, representing “≥4 h”, “3 h”, “2 h”, “1 h”, “<1 h”, and “never”, respectively; (4) “Numbers of online overnight in the past thirty days”, the answers were scored 1–5, representing “≥4 times”, “3 times”, “2 times”, “1 time” and “never”, respectively. Cronbach’s alpha was 0.662.

#### Variables of the HAPA model

The items of the HAPA model variables were measured and modified in accordance with the compilation principles of Schwarzer Ralf, and related behaviors [32, 33].

Risk perception was assessed by three items (for example, “If you compare your current Internet use behaviors to that of individuals with the same gender and age, how likely do you think your academic performance will decline?”). Items were scored with 5 Likert scales (1 = very low, 5 = very high). Cronbach’s alpha range was 0.737–0.809.

Outcome expectancy was examined by five items (for example, “Reducing Internet time is beneficial for maintaining vision.”). Items were scored with 6 Likert scales (1 = completely disagree, 6 = completely agree). Cronbach’s alpha range was 0.863–0.879.

Action self-efficacy was estimated by five items (for example, “I am confident that I would go online as little as possible in my free time.”). Items were scored with 5 Likert scales (1 = completely disagree, 5 = completely agree). Cronbach’s alpha range was 0.809–0.876.

Intention was assessed by three items (for example, “I plan to reduce my Internet use time in the next month.”). The item was scored with 6 Likert scales (1 = completely disagree, 6 = completely agree). Cronbach’s alpha range was 0.896–0.912.

Planning was determined by three items (for example, “I have made a plan for controlling the time spent online every week.”). Items were scored with 6 Likert scales (1 = completely disagree, 5 = completely agree). Cronbach’s alpha range was 0.932–0.940.

Maintenance self-efficacy was assessed by two items (for example, “After insisting on controlling the internet use time for one month, I can keep controlling even if one day exceeds the prescribed time.”) Items were scored with 6 Likert scales (1 = completely disagree, 6 = completely agree). Cronbach’s alpha was 0.833–0.896.

Sociodemographic characteristics and excessive Internet use behaviors were derived from the baseline survey. When fitting the HAPA model, we used risk perception, outcome expectancy, action self-efficacy, and intention from the third survey, and obtained planning, maintenance self-efficacy, and Internet use behaviors from the fifth survey.

### Data collection and analysis

We used unified questionnaires and survey process in the investigations. Before the interventions and surveys, we conducted uniform training for investigators to standardize the intervention and investigation process. Self-administered questionnaires were used to obtain the data of participants. After each investigation, interactive inspections were executed in investigators to find and rectify mistakes such as misfiled, omissions, and logic errors. To ensure the accuracy of the data, double entry was used for questionnaire data entry.

Epidata 3.1 was used to establish the database and data entry. SPSS 24.0 was used to sort and analyze the data. AMOS 21.0 was used to fit the SEM. The Maximum Likelihood (ML) method was used for parameter estimation, and the Bootstrap method was used synchronously when fitting the model. *P*-value < 0.05 was considered statistically significant.

In the descriptive analysis, the mean ± standard deviation (M ± SD) was used to describe quantitative variables and the frequency (%) was used to describe qualitative variables. The bivariate analysis was adopted to analyze the correlations between the variables of the HAPA model. The SEM was constructed based on the HAPA model and previous research results, and the model fitting was evaluated according to the criteria of χ^2^/df ≤ 5.0, GFI>0.90, CFI>0.90, TLI>0.90, NFI>0.90, RMSEA<0.08. The SEM can analyze the direct and indirect relationships between latent variables and observed variables in the longitudinal design, which fits the analysis framework of the applicability of the HAPA model. Therefore, SEM was used to explore the applicability of the HAPA model in excessive Internet use behaviors. If the result of the SEM shows good applicability, then the effectiveness of the HAPA interventions will be discussed further. In addition, the path coefficients of variables in the SEM could help to find the key variables in behavior change, and as the basis to make interventions. The Chi-square test and the t-test were used to compare the differences of HAPA variables and Internet use behaviors before and after the interventions.

## Results

### Sociodemographic characteristics and scores of the HAPA model variables

In the baseline survey, the mean (SD) age of the participants was (15.37 ± 1.31) years old, 168 (51.4%) were boys, 279 (85.3%) were in tenth grade, and 217 (66.4%) were left-behind adolescents. Table 2 illustrated the comparison results of the HAPA model variables of participants with different sociodemographic characteristics after the interventions. Action self-efficacy of females was higher than that of males (*P* < 0.05). Compared to low grade, senior students had a better performance in planning making and Internet use behaviors (*P* < 0.05). However, there was no statistical difference between left-behind and non-left-behind adolescents in the scores of the HAPA model variables.

**Table 2:** scores of the HAPA model variables in different sociodemographic characteristics (*n* = 327)

	Left-behind status		Gender		Grade	
	yes	no	male	female	seventh	tenth
Outcome expectancy	4.85 ± 0.79	4.84 ± 0.87	4.80 ± 0.81	4.90 ± 0.82	4.81 ± 1.02	4.85 ± 0.78
Risk perception	3.22 ± 0.89	3.16 ± 0.85	3.16 ± 0.88	3.24 ± 0.87	3.01 ± 0.96	3.23 ± 0.86
Action self-efficacy ^b*^	2.95 ± 0.87	2.99 ± 0.97	2.86 ± 0.98	3.08 ± 0.81	2.99 ± 1.17	2.96 ± 0.85
Intention	4.18 ± 1.23	4.07 ± 1.27	4.03 ± 1.25	4.25 ± 1.23	3.88 ± 1.65	4.19 ± 1.15
Planning^c*^	3.62 ± 1.41	3.83 ± 1.40	3.54 ± 1.45	3.84 ± 1.34	3.13 ± 1.58	3.78 ± 1.36
Maintenance self-efficacy	4.13 ± 1.11	4.19 ± 1.10	4.15 ± 1.08	4.15 ± 1.14	3.96 ± 1.26	4.18 ± 1.08
Internet use behaviors^a, b**, c**^	15.50 ± 3.43	15.73 ± 3.64	14.95 ± 3.48	16.23 ± 3.41	14.13 ± 3.42	15.82 ± 3.45

The t-test was used to test for the differences between groups; ^*^P < 0.05, ^**^P < 0.01

^a^Internet use behaviors were obtained by adding up four items about Internet use

^b^means that different gender adolescents have statistically different scores in this variable

^c^means that different grade adolescents have statistically different scores in this variable

### Applicability of the HAPA model in internet use behaviors

#### Correlation analysis of the HAPA model variables

The results of the correlation analysis showed that there was no statistical correlation between outcome expectancy and risk perception, maintenance self-efficacy, as well as Internet use behaviors, while other variables were all correlated (*P* < 0.05) (Table 3). Risk perception was negatively related to action self-efficacy, intention, planning, maintenance self-efficacy, and Internet use behaviors (*P* < 0.01). The correlation coefficients between other variables were positive (*P* < 0.05).

**Table 3:** Correlation analysis of the HAPA model variables(*r, n* = 327)

	Outcome expectancy	Risk perception	Action self-efficacy	Intention	Planning	Maintenance self-efficacy	Internet use behaviors
Outcome expectancy	1						
Risk perception	0.107	1					
Action self-efficacy	0.231^***^	−0.205^***^	1				
Intention	0.258^***^	−0.200^***^	0.637^***^	1			
Planning	0.122^*^	−0.184^**^	0.317^***^	0.361^***^	1		
Maintenance self-efficacy	0.064	−0.176^***^	0.257^***^	0.235^***^	0.449^***^	1	
Internet use behaviors	0.096	−0.178^**^	0.266^***^	0.353^***^	0.414^***^	0.205^***^	1

Pearson correlation was used to analyze the correlation between two variables; ^*^P < 0.05, ^**^P < 0.01, ^***^P < 0.001

#### Model fitting and analysis results

**Fig. 1:**
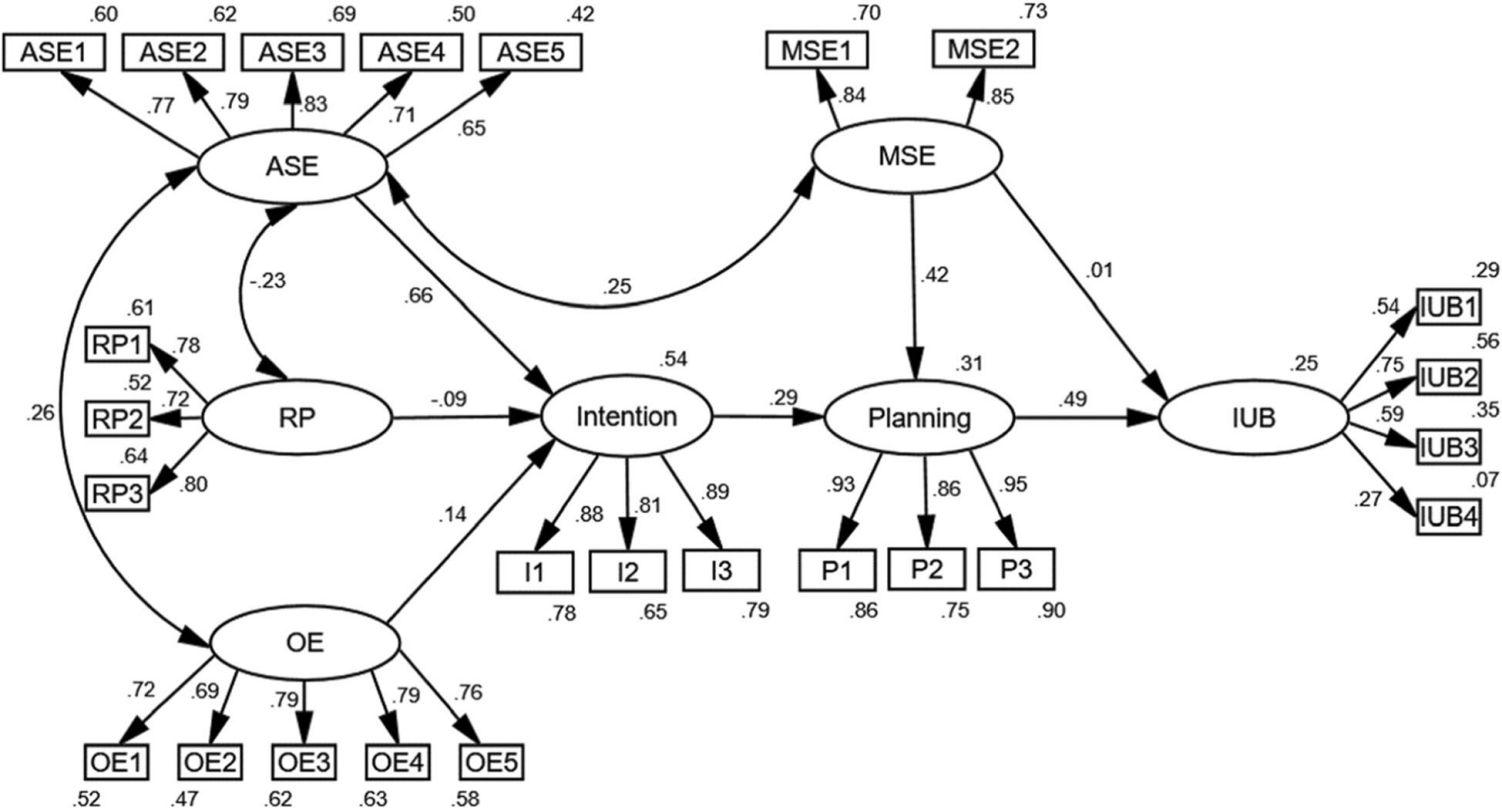
Standardized Path Coefficient of the HAPA model of Internet use behaviors. Note: ASE: action self-efficacy; RP: risk perception; OE: outcome expectancy; MSE: maintenance self-efficacy; IUB: Internet use behaviors

The initial model was obtained by adjusting action self-efficacy and risk perception, outcome expectancy, as well as maintenance self-efficacy, all of which were found relevant in previous studies [20, 34]. The modified model (model 1) was obtained by appropriate adjustment according to the modification index (MI) (Fig. 1). The fitting indices of model 1 were as follows: χ^2^/df = 2.066, GFI = 0.889, CFI = 0.938, TLI = 0.928, IFI = 0.938, RMSEA = 0.057, indicating that the model was acceptable. The results of group analysis of SEM illustrated that model 1 was applicable to the Internet use behaviors of rural adolescents with different gender, grade, and left-behind status.

The path coefficients of model 1 showed that except for the path from risk perception to intention and the path from maintenance self-efficacy to Internet use behaviors, other paths were statistically significant (*P* < 0.05). The indirect effects of model 1 were presented in Table 4. The results showed that the 95% confidence interval of the intermediate paths with risk perception as an independent variable contained 0, while other intermediate paths did not contain 0. Generally, all variables could explain 25% of Internet use behaviors in model 1.

**Table 4:** The path coefficients of the HAPA model (model 1, *n* = 327)

		Standardized coefficient (95% CI)	Unstandardized coefficient (standard error)	*P*-value
Direct effects	Action self-efficacy→ Intention^**^	0.660 (0.525, 0.779)	0.904 (0.104)	0.001
	Risk perception→ Intention	−0.088(− 0.193, 0.023)	−0.124 (0.077)	0.114
	Outcome expectancy→ Intention^*^	0.144 (0.021, 0.265)	0.224 (0.093)	0.023
	Intention→ Planning^**^	0.293 (0.176, 0.413)	0.332 (0.069)	0.001
	Maintenance self-efficacy→ Planning^**^	0.424 (0.272, 0.560)	0.584 (0.110)	0.001
	Maintenance self-efficacy→ Internet use behaviors	0.008(−0.180, 0.189)	0.006 (0.073)	0.976
	Planning→ Internet use behaviors^**^	0.494 (0.346, 0.643)	0.274 (0.051)	0.001
Indirect effects	Outcome expectancy→ Intention→ Planning^*^	0.042 (0.006, 0.088)	0.074 (0.037)	0.023
	Outcome expectancy→ Intention→ Planning→ Internet use behaviors^*^	0.021 (0.003, 0.048)	0.020 (0.011)	0.023
	Risk perception→ Intention→ Planning	−0.026(−0.063, 0.006)	− 0.041 (0.028)	0.114
	Risk perception→ Intention→ Planning→ Internet use behaviors	−0.013(− 0.033, 0.003)	−0.011 (0.008)	0.114
	Action self-efficacy→ Intention→ Planning^**^	0.193 (0.116, 0.279)	0.300 (0.068)	0.001
	Action self-efficacy→ Intention→ Planning→ Internet use behaviors^**^	0.095 (0.048, 0.154)	0.082 (0.025)	0.001
	Intention→ Planning→ Internet use behaviors^**^	0.145 (0.072, 0.231)	0.091 (0.027)	0.001
	Maintenance self-efficacy→ Planning→ Internet use behaviors^**^	0.209 (0.117, 0.314)	0.160 (0.044)	0.001

^*^P < 0.05, ^**^P < 0.01. The starting of the arrows were independent variables, and the end of the arrows were dependent variables

### Effectiveness of interventions based on the HAPA model

The results in Table 5 displayed that outcome expectancy, intention, and maintenance self-efficacy were significantly improved in the experimental school. The rate of excessive Internet use was declined from 100 to 70%, meanwhile, all Internet use behaviors have been declined to some extent, and there were statistically significant differences in daily Internet time on weekends and daily game time (Table 6).

**Table 5:** The HAPA model variables in the control and the experimental group

	Experimental group (*n* = 327)				Control group (*n* = 448)			
	Before	After	Incremental effect	*P* value	Before	After	Incremental effect	*P* value
Risk perception	3.29 ± 0.97	3.20 ± 0.88	−0.09	0.122	3.02 ± 1.04	2.77 ± 0.98	−0.25	< 0.001
Outcome expectancy	3.86 ± 0.72	4.85 ± 0.82	0.99	< 0.001	3.84 ± 0.76	4.56 ± 1.10	0.72	< 0.001
Action self-efficacy	2.92 ± 0.92	2.97 ± 0.90	0.05	0.388	2.74 ± 1.00	2.83 ± 0.90	0.09	0.09
Intention	3.55 ± 1.18	4.14 ± 1.24	0.59	< 0.001	3.71 ± 1.16	4.09 ± 1.39	0.38	< 0.001
Planning	3.70 ± 1.40	3.69 ± 1.41	−0.01	0.851	3.55 ± 1.47	3.55 ± 1.59	0	0.961
Maintenance self-efficacy	3.43 ± 1.27	4.15 ± 1.11	0.72	< 0.001	3.53 ± 1.33	3.94 ± 0.82	0.41	< 0.001

The data of risk perception, outcome expectancy, action self-efficacy, and intention were derived from the baseline survey and the third survey. The data of planning and maintenance self-efficacy came from the third survey and the fifth survey. In the comparison of HAPA variables, the average scores of items were compared

The t-test was used to test the differences before and after interventions

**Table 6:** Internet use behaviors of intervened participants in three surveys (*n* = 327)

Variables	Baseline survey N (%)	The third survey N (%)	The fifth survey N (%)	*P*-value
Excessive Internet user	327 (100.0%)	256 (78.29%)	229 (70.0%)	
Internet use behaviors^*^				
Average daily Internet time from Monday to Friday ≥ 2 h	169 (51.7%)	179 (54.74%)	155 (47.4%)	0.258
Average daily Internet time on weekends ≥ 4 h	187 (57.2%)	176 (53.82%)	128 (39.1%)	< 0.001
Usually daily game time ≥ 2 h	167 (51.1%)	136 (41.59%)	115 (35.2%)	< 0.001
Have been online overnight at least once in the past 30 days	75 (22.9%)	53 (16.21%)	64 (19.5%)	0.289

The Chi-square test was used to test the differences between the baseline and the fifth survey

^*^The *P*-values of Internet use behaviors were obtained from the results of the comparisons between the baseline survey and the fifth survey

## Discussion

The results of this study illustrated the HAPA model has good applicability in Internet use behaviors, moreover, interventions based on the HAPA model can effectively improve social-cognitive factors of the excessive Internet use behaviors of Chinese rural adolescents. After the interventions, the rate of excessive Internet users reduced by 30%. Among participants, the rate of average daily Internet time on weekends≥4 h dropped from 57.2 to 39.1%, and the rate of usually daily game time ≥ 2 h reduced from 51.1 to 35.2%. Although no improvement was observed in average daily Internet time on weekdays≥2 h and online overnight in the past 30 days, the rates after intervention did not increase. Meanwhile, the interventions improved outcome expectancy, intention, and maintenance self-efficacy of rural adolescents.

### Applicability of the HAPA model in internet use behaviors

The results of SEM identified that the HAPA model has good applicability in rural adolescents’ Internet use behaviors. In model 1, most paths had been effectively verified, apart from risk perception to intention, and maintenance self-efficacy to Internet use behaviors. We found that there was only an indirect impact between maintenance self-efficacy and Internet use behaviors, while the direct effect was not observed. It demonstrated that for Internet use behaviors of adolescents, the role of maintaining self-efficacy was to promote planning convert into behaviors and to maintain the implementation of planning. Further, the strong correlation between action self-efficacy and maintenance self-efficacy had been verified [20].

Different from the HAPA hypothesis, this study did not find the association between risk perception and intention similarly to other studies [35–37]. It might be related to the description of the items. In this study, the items of risk perception were described as “If you compare your current Internet use behaviors to that of individuals with the same gender and age, how likely do you think …”.They might not deem that their Internet use behaviors were worse than that of same-gender peers, nor they would encounter the risks mentioned in the items [38]. Risk perception having little effect on intention and behaviors have been reported by previous studies applying the HAPA model in different behaviors [20, 36, 37, 39]. Behaviors such as taking medicines and vaccination could reduce the risk of diseases, closely associated with risk perception. On the contrary, the risks brought by daily lifestyles (including Internet use behaviors, physical activity, and diet) were not easy to be perceived, which may attenuate the impact of risk perception on behaviors in the general population. It was difficult for adolescents to perceive the risk of excessive Internet use, for whom the comprehension and perception abilities were slightly lower than those of adults.

### Effectiveness of interventions based on the HAPA model

Previous school-based studies were designed to improved Internet use behaviors of urban adolescents. The studies focused on health education courses have positive effects on game time, while no impact on daily online time [17, 40]. Different from urban adolescents, Internet use behaviors of rural adolescents have not received enough attention and lacked targeted intervention measures. This may be the reason when facing the rapidly developing Internet rural adolescents lack sufficient correct knowledge and lack of self-efficacy, which may lead to excessive Internet use. Therefore, our interventions focused on enhancing the cognitions about Internet use. However, as time goes by, the academic pressure faced by participants gradually increases, which will also cause a natural reduction in online time. In this study, the control school strengthened the academic management during the study period, which caused the participants in the control school to face higher academic pressures than those in the experimental school. Meanwhile, it is the primary reason for cannot directly compare the changes in Internet use behaviors between the experimental school and the control school. Nonetheless, by comparing the changes of HAPA variables before and after interventions in two schools, the results revealed that interventions based on the HAPA model can effectively enhance outcome expectancy, intention, and maintenance self-efficacy, which were the most important variables for reducing online time. Although we cannot affirm that the reduction of Internet use in the experimental school was entirely due to interventions, it is deemed that interventions based on the HAPA model can promote cognitive changes and thus intervene the Internet use behaviors.

In this study, the changes in outcome expectancy, intention, and maintenance self-efficacy were key indicators to the reduction of Internet use. Previous studies have claimed that outcome expectancy is the strongest predictor of intention [37, 41, 42]. Adolescents are inclined to generate the intention of reducing online time based on the awareness that reduction of online time could bring benefits for themselves such as improving the eyesight and academic performance [43, 44].

Meanwhile, intention is the most predictable factor for future behaviors [20]. The generation of intentions is a prerequisite for planning and transforming planning into behaviors. According to the HAPA model theory, behavior change needs a long-time process for excessive Internet users. Before reducing Internet use time, they need to undergo cognitive enhancement, intention formation, plan formulation and implementation, which meaning that only a few participants would reduce Internet time before planning interventions. These participants may have a good capability of planning formulation and implementation, and they may find it difficult to answer questions about the intention to reduce online time. However, by comparing the scores of the intention in the first and the third survey, we think that these participants did not influence the overall response and the interventions targeted intention were effective. Besides, maintenance self-efficacy is fundamental in the production of behaviors. Individuals with high maintenance self-efficacy are more likely to convert planning into behaviors and believe that they can overcome the potential obstacles when adopting aim behavior [45]. Interventions based on the HAPA model lead to changes in behaviors by enhancing the cognition of these three key variables of the participants. Interestingly, there were certain differences in the HAPA variable scores of adolescents with different genders and different grades after the intervention, which denotes differences in their perception corresponding to targeted intervention. It meant that boys should strengthen the interventions of action self-efficacy, and younger adolescents are supposed to focus on planning.

Unexpectedly, there was no significant improvement in action self-efficacy and planning. One possible explanation may be the interventions focused on health education were low-intensity for action self-efficacy and planning [43]. Moreover, Bandura (1977) has proposed that four sources of information influenced self-efficacy, including verbal persuasion, performance attainment, vicarious experiences, and affective or psychological states [44]. The interventions of action self-efficacy in this study centered on increasing participants’ vicarious experiences and verbal persuasion. While performance attainment, especially previous successful experiences, was considered the most prominent source of self-efficacy according to Bandura [44]. Future interventions can offer adolescents successful experiences through scenario simulation, role-playing, etc., to increase action self-efficacy of the target behaviors.

One of the interventions in this study was to increase physical exercise time by providing sports equipment, thereby reducing online time. Although there was no investigation on the specific use of sports equipment, through qualitative interviews with school teachers, it was found that these sports equipment were popular among participants, and often used after classes. This showed that increasing physical exercise time can indirectly intervene with excessive Internet use behaviors.

It is worth mentioning that the ideal results of interventions should be obtained from the comparison between the experimental school and the control school. However, because of the heavy academic pressure, the control school had added a strict system of residence in the research process, and forbidden students to bring mobile phones and any other electronic products into campus. And students can go home for only 2-day holidays each month. While the experimental school didn’t have such rules. Therefore, it is impossible to directly compare the change degree of excessive Internet use in the experimental school with the control school. Nevertheless, it could be seen that the social-cognitive factors from HAPA in the experimental school were improved more than those in the control school.

### Limitations and strengths

There were some limitations to this study. First, the data came from the self-reports of the participants, and there might be reporting bias. Second, previous studies confirmed that planning could be divided into action planning and coping planning. Action planning is important for the beginning of behaviors, and coping planning is crucial to the maintenance of behaviors [46]. However, this study did not refine them. Third, in the research process, the subjects of the control school were affected by the external environment such as the strict management system, which made it difficult to directly compared the status of excessive Internet use in the experimental school with the control school. This is the biggest weakness of this study. It is hoped that future researches can improve this deficiency in this study and obtain more realistic results to illustrate the effectiveness of interventions based on the HAPA model.

Despite these limitations, the strengths of this study could not be ignored. This study applied the HAPA model to intervene in adolescents’ excessive Internet use behaviors, which could provide new ideas for understanding the cognitive process of Internet use behaviors in adolescents. Further, longitudinal data in this study were used for analysis to better illustrate the continuous process of cognitive, intention, and behavioral change. Since the transformation of intention, planning and behaviors takes time, the longitudinal design was able to describe the transformation process. Besides, targeted intervention measures were formulated based on the HAPA model, and a positive effect had been obtained in the Internet use behaviors of adolescents. This study not only verified the effectiveness of the HAPA model in guiding behavioral interventions but also proposed several effective measures to reduce rural adolescents’ excessive Internet use behaviors.

## Conclusion

This study applied the HAPA model as the theoretical framework to examine its applicability and effectiveness for reducing excessive Internet use behaviors in Chinese rural adolescents. The results of confirmatory research revealed that the HAPA model was effectively verified in Internet use behaviors in rural adolescents. The results of intervention research suggested that interventions based on the HAPA model could effectively reduce excessive Internet use behaviors in rural adolescents, mainly through the enhancement of outcome expectancy, intention, and maintenance self-efficacy.

## Supplementary Information

**Additional file 1: Appendix table 1.** The comparison between the control group and the experiment group before interventions.

## Data Availability

The data and materials used in this paper are not public without the permission of Sichuan University. If there is a reasonable request, please contact the corresponding author for more data.

## Abbreviations

HAPA: Health Action Process Approach model
SEM: Structural equation model
CNNIC: China Internet Network Information Center
CBT: Cognitive-behavioral therapy
ML: Maximum Likelihood
M ± SD: Mean ± standard deviation
MI: Modification index
ASE: Action self-efficacy
RP: risk perception
OE: Outcome expectancy
MSE: Maintenance self-efficacy
IUB: Internet use behaviors
CI: Confidence interval
