# Supplementary material for: Application of the health action process approach model for reducing excessive internet use behaviors among rural adolescents in China: a school-based intervention pilot study
Source: BMC Public Health. 2021 May 26;21:986. doi: 10.1186/s12889-021-10999-z (PMC8152115; doi:10.1186/s12889-021-10999-z)
Supplement: Supplementary file 1 — Additional file 1: Appendix table 1. The comparison between the control group and the experiment group before interventions. [file 12889_2021_10999_MOESM1_ESM.docx]

**Appendix table 1.** The comparison between the control group and the experiment group before interventions

|  | Control group | Experiment group | P-value |
| --- | --- | --- | --- |
| Age | 15.32±1.24 | 15.37±1.31 | 0.645 |
| Gender |  |  | 0.675 |
| Male | 237 | 168 |  |
| Female | 211 | 159 |  |
| Grade |  |  | 0.488 |
| Seventh | 58 | 48 |  |
| Tenth | 390 | 279 |  |
| Left-behind status |  |  | <0.001 |
| No | 217 | 110 |  |
| Yes | 231 | 217 |  |
